# Triglyceride: A mediator of the association between waist-to-height ratio and non-alcoholic fatty liver disease: A second analysis of a population-based study

**DOI:** 10.3389/fendo.2022.973823

**Published:** 2022-10-31

**Authors:** Haofei Hu, Yong Han, Yufei Liu, Mijie Guan, Qijun Wan

**Affiliations:** ^1^ Department of Nephrology, Shenzhen Second People’s Hospital, Shenzhen, Guangdong, China; ^2^ Department of Nephrology, The First Affiliated Hospital of Shenzhen University, Shenzhen, Guangdong, China; ^3^ Shenzhen University Health Science Center, Shenzhen University, Shenzhen, Guangdong, China; ^4^ Department of Emergency, Shenzhen Second People’s Hospital, Shenzhen, Guangdong, China; ^5^ Department of Emergency, The First Affiliated Hospital of Shenzhen University, Shenzhen, Guangdong, China; ^6^ Department of Neurosurgery, Shenzhen Second People’s Hospital, Shenzhen, Guangdong, China; ^7^ Department of Neurosurgery, The First Affiliated Hospital of Shenzhen University, Shenzhen, Guangdong, China

**Keywords:** Waist-to-height ratio, triglyceride, non-alcoholic fatty liver disease, mediation analysis, logistic regression

## Abstract

**Objective:**

Increasing evidence suggests that an increased waist-to-height ratio (WHtR) may increase the risk of non-alcoholic fatty liver disease (NAFLD). Whether this association is due to WHtR itself or mediated by WHtR-associated increases in triglyceride (TG) is uncertain. On that account, our research aims to disentangle these relationships.

**Methods:**

In this cross-sectional study, 14251 participants who participated in the medical examination program were consecutively and non-selectively collected in Murakami Memorial Hospital in Japan from 2004 to 2015. The independent and dependent variables were WHtR and NAFLD, respectively. Triglyceride was the mediating factor. The correlation between WHtR, TG, and NAFLD risk factors was examined using spearman correlation analysis. The association between WHtR or TG and NAFLD was examined using multiple logistic regression. In order to determine whether TG mediated the association between WHtR and NAFLD, a mediation analysis was performed.

**Results:**

The mean age of the included individuals was 43.53 ± 8.89 years old, and 7411 (52.00%) were male. The mean WHtR and TG were 0.46 ± 0.05, 0.89 ± 0.63, respectively. The prevalence rate of NAFLD was 2507 (17.59%). Individuals with NAFLD had significantly higher levels of WHtR and TG than those without NAFLD (P<0.05). After adjusting covariates, the multivariate linear regression analysis showed that WHtR was positively associated with TG. That was, for every 0.1 increase in WHtR, TG increased by 0.226mmol/L (β=0.226, 95%CI: 0.206, 0.247). Multiple logistic regression analysis indicated that WHtR (OR=8.743, 95%CI: 7.528, 10.153) and TG (OR=1.897, 95%CI: 1.732, 2.078) were positively associated with NAFLD. The mediation analysis showed that WHtR had a direct, significant effect on NAFLD (β=0.139, 95%CI: 0.126, 0.148), and TG partially mediated the indirect effect of WHtR on NAFLD (β=0.016, 95% CI: 0.013-0.019). TG contributed to 10.41% of WHtR-related NAFLD development.

**Conclusion:**

Findings suggest a mediation link between WHtR and TG and the risk of NAFLD. The significance of TG as a mediator deserves recognition and consideration.

## 1 Background

Non-alcoholic fatty liver disease (NAFLD) involves a spectrum of liver injury processes, from simple hepatic steatosis to non-alcoholic steatohepatitis (NASH), which can further progress to cirrhosis, liver failure, and hepatocellular carcinoma (1). NAFLD has become a global public health problem that endangers human health, affecting approximately one-quarter of adults worldwide (2, 3). With an estimated 25-45% prevalence in western countries and 29.62% in Asia, NAFLD is now the most prevalent chronic liver disease (4–7). And the incidence and prevalence of NAFLD are rapidly increasing (8, 9). NAFLD is also closely related to cardiovascular disease (10), type 2 diabetes (11), and chronic kidney disease (12). Furthermore, NAFLD patients had a relatively high mortality rate compared to the general population (13). Therefore, finding the risk factors and mechanisms of NAFLD is necessary to prevent the disease.

A large amount of hepatic triglycerides are accumulated during the onset of NAFLD, as well as insulin resistance (IR) (14). NAFLD pathophysiology involves the accumulation of neutral lipids, predominantly triglycerides (TG), in the liver (15). Excessive accumulation of TG in hepatocytes is a critical factor in NAFLD (16). Studies have shown that hypertriglyceridemia (HTG) and IR are known risk factors for NAFLD development (17–19). A part of the accumulation of TG can be attributed to obesity (20). Waist circumference (WC) and body mass index (BMI) are currently the most widely used anthropometric indicators that assess obesity globally, as well as the most significant risk factors for NAFLD (21–23). Recent studies have identified the waist-to-height ratio (WHtR) as an effective method of assessing the risk of central obesity, type 2 diabetes, and hypertension among others (24–27). The WHtR has also been found to be a better indicator of NAFLD risk and severity and a more sensitive diagnostic tool than WC and BMI (28–31).

However, the pathway of the contribution of the WHtR to the increased risk of NAFLD is still unclear. Whether TG plays a role in this process is also unclear. Our aim in this analysis was to use a mediation approach with WHtR as a major determinant of NAFLD and TG as a potential mediator in the relation between adherence to a WHtR and NAFLD.

## 2 Methods

### 2.1 Study design

This study was a second cross-sectional study using the data obtained from NAGALA (NAfld in the Gifu Area, Longitudinal Analysis) database established by Murakami Memorial Hospital in Japan. We set the baseline WHtR as the target-independent variable and NAFLD (dichotomous variable: 1=NAFLD, 0= non-NAFLD) as the dependent variable. Simultaneously, we set TG as the mediating factor.

### 2.2 Data source

Raw data were obtained from the DATADRYAD database (https://datadryad.org/stash/ ) provided by Okamura, Takuro, et al. (2019). Data from: Ectopic fat obesity presents the greatest risk for incident type 2 diabetes: a population-based longitudinal study, Dryad, Dataset, https://doi.org/10.5061/dryad.8q0p192. The Dryad terms of service allowed other researchers to use the data for secondary analyses without compromising the authors’ rights.

### 2.3 Study population

Participants were collected consecutively from Murakami Memorial Hospital in Japan to minimize selection bias. Their identity information was encoded into an untraceable code to ensure participants’ privacy. The clinical data of participants were acquired and stored in the electronic data capture system named NAGALA database. This study was performed according to the Declaration of Helsinki, and the clinical research ethics committee approved all procedures involving humans at Murakami Memorial Hospital. All participants involved in this study have signed informed consent after explaining the study.

**Figure 1:**
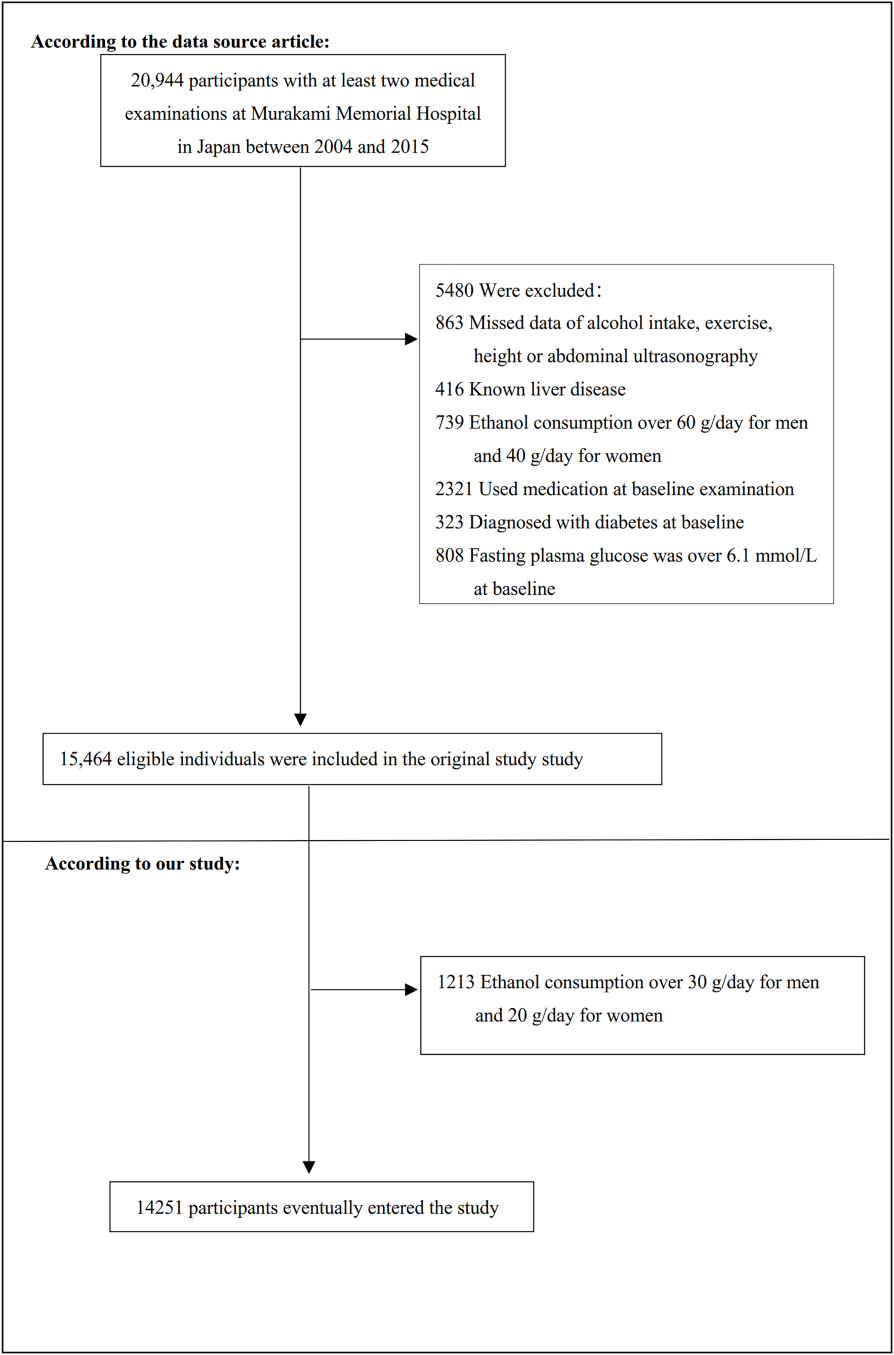
Flowchart of study participants. Figure 1 showed the inclusion of participants. The eligibility of 15,464 participants was assessed in the original study. We excluded patients with ethanol consumption over 30 g/day for men and 20 g/day for women (n=1213). The final analysis included 14251 subjects in the present study.

The study initially included 20944 participants; afterward, 6693 participants were excluded, and 14251 persons were left for data analysis(see flowchart for details in **Figure 1**). All clinical steps in the present study followed the Strobe statement (32). Inclusion criteria: participants who participated in physical exams between 2004 and 2015, completed at least two physical exams. Exclusion criteria included (1): participants diagnosed with type 2 diabetes (n=323) or with fasting plasma glucose (FPG) was over 6.1 mmol/L (n=808) (2); participants with known liver disease, such as hepatitis B or C virus (n=416) (3); anyone who took medication, including antihypertensive, antiglycemic and lipid-lowering drugs (n=2321) (4); people with excessive drinking habits (more than 20 grams daily for women and more than 30 grams daily for men)(n=1952) (5); participants with a missed value of covariates, including abdominal ultrasonography, exercise, alcohol intake or laboratory variables (n=863) (33).

### 2.4 Variables

#### 2.4.1 Waist-to-height ratio

We recorded the WHtR as a continuous variable and obtained its information at baseline. The detailed process of measuring WHtR was described as follows: WHtR = waist (cm) divided by height (cm).

#### 2.4.2 Triglyceride

After a night of fasting, venous blood was collected for TG testing. HTG refers to serum TG levels ≥1.7 mmol/L (34). We divided the participants into two groups, those with hypertriglyceridemia and those without hypertriglyceridemia.

#### 2.4.3 Diagnosis of NAFLD

An abdominal ultrasound was used to assess NAFLD, and gastroenterologists without knowledge of the participants’ personal information, reviewed the ultrasound images. The final diagnosis was made based on the evaluation of four ultrasound findings: liver brightness, liver and kidney echo contrast, vessel blurring, and depth attenuation (35).

#### 2.4.4 Covariates

The present study selected covariates based on clinical experience and published literature references. Therefore, we use the following variables as covariates based on the above principles (1): continuous variables: age, systolic blood pressure(SBP), total cholesterol(TC), gamma-glutamyl transferase(GGT), diastolic blood pressure (DBP), alanine aminotransferase(ALT), FPG, ethanol consumption, aspartate aminotransferase(AST), hemoglobin A1c (HbA1c) (2); categorical variables: the habit of exercise, smoking status, and sex.

As mentioned in the NAGALA study, this study’s clinical baseline information was collected through a standardized self-administered questionnaire, including smoking and alcohol habits, medical history, and physical activity. Participants’ average weekly ethanol intake assessed ethanol consumption during the preceding month. Participants’ recreational and sports activities were investigated to classify participants as non-exercise or regular exercisers (36). Regular exercisers were defined as those who reported any exercise more than once a week (37). BMI (kg/m^2^) = body weight (kg)/height^2^ (m^2^). After a night of fasting, venous blood was collected for hematological indicators testing, including TC, GGT, ALT, AST, HbA1c, and FPG.

### 2.5 Statistical analysis

For continuous variables, the mean (standard deviation) was given for normal distribution, the median (range) for non-normal distribution, and the number (%) for categorical variables. We used the Student’s t-test (normal distribution), the χ2 (categorical variables), or the Mann-Whitney’s U-test test (non-normal distribution) to test for differences between individuals with or without NAFLD.

The correlation between WHtR and TG and potential NAFLD risk factors was explored using spearman correlation analysis. To investigate the association between WHtR and TG, three distinct models using the univariate and multivariate linear regression model were performed, including the non-adjusted model (no covariates were adjusted), minimally-adjusted model (only sociodemographic variables were adjusted, including SBP, age, ethanol consumption, sex, smoking status and habit of exercise) and fully-adjusted model (including SBP, age, ALT, sex, AST, FPG, TC, GGT, HbA1c, ethanol consumption, the habit of exercise, smoking status). Effect sizes(β) with 95% confidence intervals(CI) were recorded.

To investigate the association between WHtR or TG and NAFLD, three distinct models using the univariate and multivariate logistic regression models were performed, including the non-adjusted model, minimally-adjusted model (Model I, including SBP, age, ethanol consumption, sex, smoking status, and habit of exercise) and fully-adjusted model (including SBP, age, ALT, sex, AST, FPG, TC, GGT, HbA1c, ethanol consumption, the habit of exercise, smoking status). When analyzing the relationship between WHtR and NAFLD, we additionally adjusted for TG. Whereas when examining the relationship of TG and NAFLD, we further adjusted for WC and BMI in the equations. Effect sizes(OR) with 95% confidence intervals(CI) were recorded. After adding the covariances, we adjusted them, and the effect sizes (β or OR) changed by 10% or more (32). Also, it referred to the results of the collinearity screening. Collinearity screening showed that DBP was collinear with other variables, so DBP was not included in the multivariate linear or logistic regression equations. We converted the WHtR or TG from a continuous variable to a categorical variable based on clinical cut-off points or tertiles and calculated the P for trend, in order to verify the results whether the WHtR or TG as a continuous variable was consistent with the categorical variable.

To determine whether the effect of the treatment variable (WHtR) on the outcome variable (NAFLD) was mediated by the mediator variable (TG), a mediation analysis was conducted. Mediation analyses could quantify the total effect (association between WHtR and NAFLD), natural direct effect (total effect without the influence of TG), and natural indirect effect (effect of WHtR on NAFLD attributed to TG). To measure the adjusted mediation effect, the covariates ALT, age, SBP, HbA1c, AST, sex, TC, GGT, FPG, ethanol consumption, smoking status, and habit of exercise were adjusted for in the mediation analysis through three different models.

We used stratified mediation analysis to explore the results’ robustness in various subgroups (sex, SBP, ethanol consumption, DBP, the habit of exercise, BMI, age, and smoking status). Firstly, we converted the continuous variable SBP (<140mmHg, ≥140mmHg), DBP (<90mmHg, ≥90mmHg), age (<30, 30 to 40, 40 to 50, 50 to 60, ≥60) (38), ethanol consumption(=0g/week, >0g/week), BMI (<25kg/m^2^, ≥25 kg/m^2^) (39) to a categorical variable based on the clinical cut point. Secondly, in addition to the stratification factor itself, we adjusted each stratification for all factors (ALT, age, SBP, HbA1c, AST, sex, TC, GGT, FPG, ethanol consumption, smoking status, and habit of exercise).

Modeling was carried out using statistical packages from the R (http://www.r-project.org , The R Foundation ) and EmpowerStats packages (http://www.empowerstats.com , X&Y Solutions, Inc, Boston, MA). Statistical significance was defined as a P value less than 0.05 (two-sided).

## 3 Results

### 3.1 Participants’ baseline characteristics

Baseline characteristics were depicted in **Table 1**. Of the 14251 included participants, the mean age was 43.53 ± 8.89 years, and 52.00% were males. The mean WHtR and TG were 0.46 ± 0.05, 0.89 ± 0.63, respectively. The prevalence rate of NAFLD was 2507 (17.59%). Individuals were divided into two groups based on liver ultrasonography: the NAFLD group (n=2507) and the non-NAFLD group (n=11744). In the comparison based on the non-NAFLD, the higher value or proportion of age, AST, BMI, ALT, height, WC, GGT, WHtR, HbA1c, SBP, TC, TG, males, DBP, FPG, ex-smokers, and current smokers were detected in the NAFLD group (**Figure S1**). In comparison, the lower value and proportion of high-density lipoprotein cholesterol (HDL-c), regular exercisers, females, and non-smokers were observed.

**Table 1:** The Baseline Characteristics of participants.

Variable	Non-NAFLD	NAFLD	P-value
**Participants**	11744	2507	
**Age, years**	43.27 ± 8.99	44.78 ± 8.33	<0.001
**Ethanol consumption, g/week**	1.00 (0.00-36.00)	1.00 (0.00-36.00)	0.003
**BMI, kg/m^2^**	21.33 ± 2.61	25.50 ± 3.13	<0.001
**WC, cm**	74.09 ± 7.92	85.98 ± 7.79	<0.001
**Height, cm**	164.11 ± 8.44	168.03 ± 7.90	
**WHtR**	0.45 ± 0.04	0.51 ± 0.05	<0.001
**ALT, IU/L**	15.00 (12.00-20.00)	27.00 (20.00-39.00)	<0.001
**A**S**T, IU/L**	17.35 ± 8.14	22.35 ± 9.79	<0.001
**GGT, IU/L**	14.0 (11.00-18.00)	23.0 (16.00-33.00)	<0.001
**HDL**-c**, mmol/L**	1.52 ± 0.40	1.18 ± 0.29	<0.001
**TC, mmol/L**	5.06 ± 0.85	5.44 ± 0.87	<0.001
**TG, mmol/L**	0.65 (0.45-0.95)	1.24 (0.87-1.80)	<0.001
**HbA1c, %**	5.15 ± 0.31	5.30 ± 0.33	<0.001
**FPG, mmol/L**	5.09 ± 0.40	5.39 ± 0.36	<0.001
**SBP, mmHg**	111.91 ± 14.02	123.41 ± 14.83	<0.001
**DBP, mmHg**	69.69 ± 9.85	77.81 ± 10.19	<0.001
**Male, n(%)**	5382 (45.83%)	2029 (80.93%)	<0.001
**Regular exercisers, n(%)**	2093 (17.82%)	377 (15.04%)	<0.001
**Smoking status, n(%)**			<0.001
Non-smoker	7561 (64.38%)	1185 (47.27%)	
Ex-smoker	1920 (16.35%)	639 (25.49%)	
Current smoker	2263 (19.27%)	683 (27.24%)	

Values are n(%) or mean ± SD or medians (quartiles).

ALT, alanine aminotransferase; AST, aspartate aminotransferase; BMI, body mass index; DBP, diastolic blood pressure; FPG, fasting plasma glucose; GGT, gamma-glutamyl transferase; HbA1c, hemoglobin A1c; HDL-c, high-density lipoprotein cholesterol; SBP, systolic blood pressure; TC, total cholesterol; TG, triglyceride; WC, waist circumference; WHtR, waist-to-height ratio.

**Figure 2:**
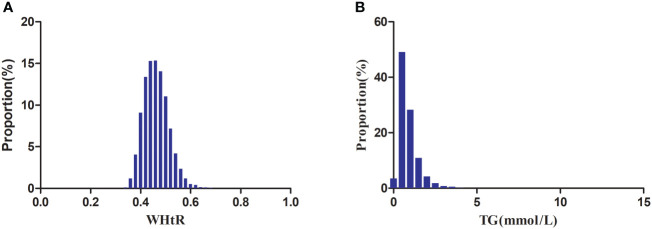
Distribution of WHtR and TG. Figure 2**(A)** showed that WHtR presented a normal distribution ranging from 0.28 to 0.788, with a mean level of 0.462. Figure 2**(B)** indicated that TG presented a skewed distribution ranging from 0.068 to 10.274, with a median level of 0.723.

**Figure 2** showed the distribution of WHtR and TG. WHtR presented a normal distribution ranging from 0.28 to 0.788, with a mean level of 0.462. TG presented a skewed distribution ranging from 0.068 to 10.274, with a median level of 0.723.

### 3.2 The prevalence rate of NAFLD

**Figure 3:**
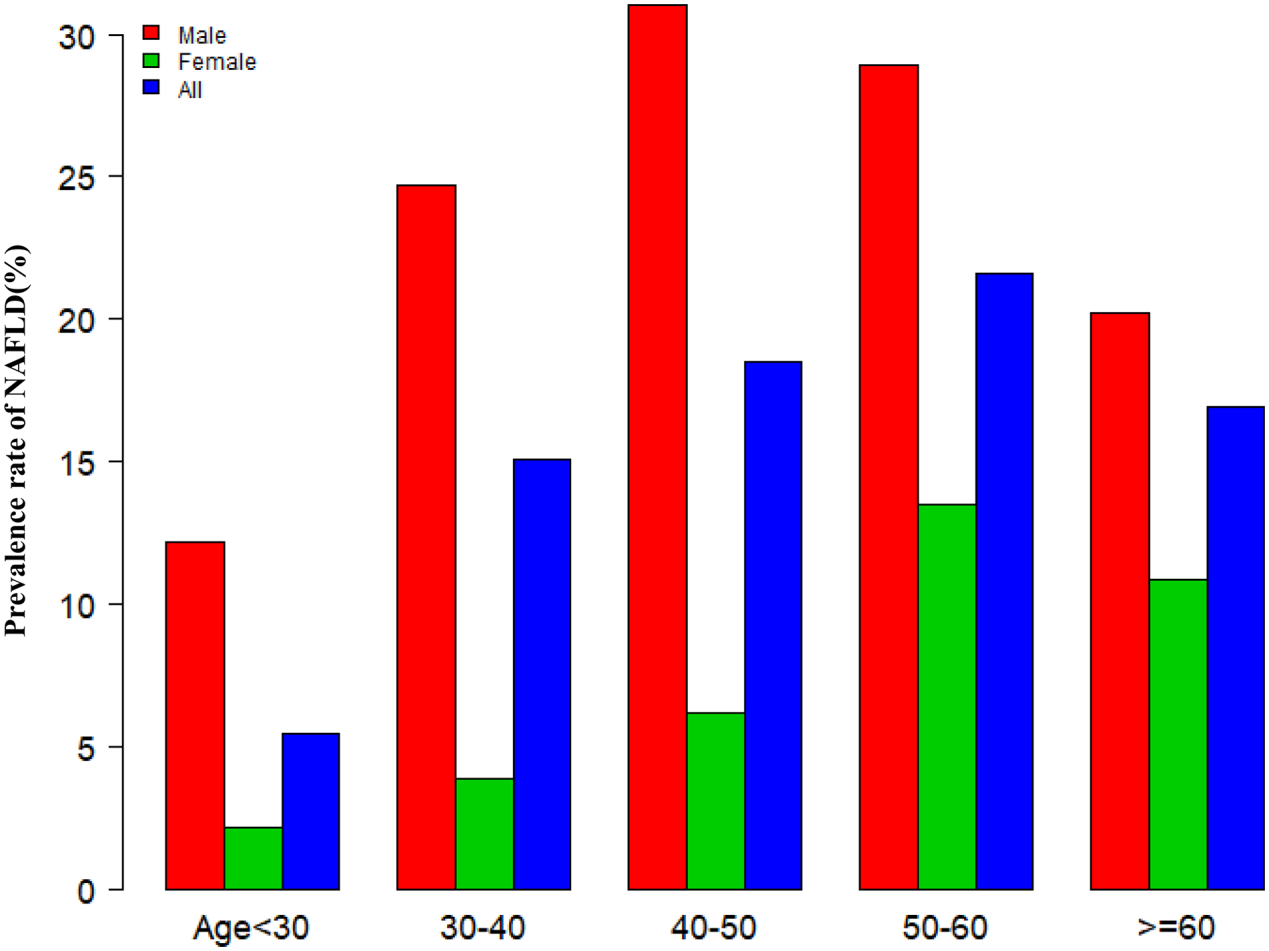
NAFLD prevalence of age stratification by 10 intervals. Figure 3 showed that across the 10 age stratifications, male subjects had higher prevalence rates of NAFLD than female subjects, regardless of age group. It also found that the prevalence of NAFLD increased with age in both male(except age≥50 years) and female(except age≥60 years) subjects.

**Figure 3** showed that across the 10 age stratifications, male subjects had higher prevalence rates of NAFLD than female subjects, regardless of age group (P<0.01). It also found that the prevalence of NAFLD increased with age in both male (except age≥50 years) and female(except age≥60 years) subjects (P for trend<0.001).

**Figure 4:**
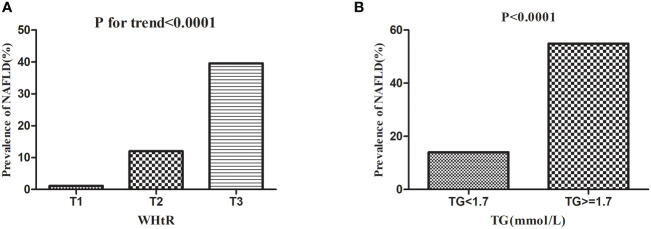
Prevalence rate of NAFLD according to the TG groups and WHtR tertiles. Figure 4 **(A)** showed that the prevalence of NAFLD increased as the WHtR group grew, and the trend test was statistically significant (P < 0.0001 for trend). Figure 4**(B)** showed that the prevalence of NAFLD was significantly higher in those with hypertriglyceridemia than those without hypertriglyceridemia (P < 0.0001).

Dividing all participants into three groups according to tertiles of WHtR, **Figure 4** showed that the prevalence of NAFLD increased as the WHtR group grew, and the trend test was statistically significant (P for trend<0.0001). When dividing the study population into two groups according to whether they had hypertriglyceridemia, the prevalence of NAFLD was significantly higher in those with hypertriglyceridemia than those without hypertriglyceridemia (P<0.0001).

### 3.3 Spearman correlation of WHtR or TG with potential NAFLDvrisk factors

**Figure 5:**
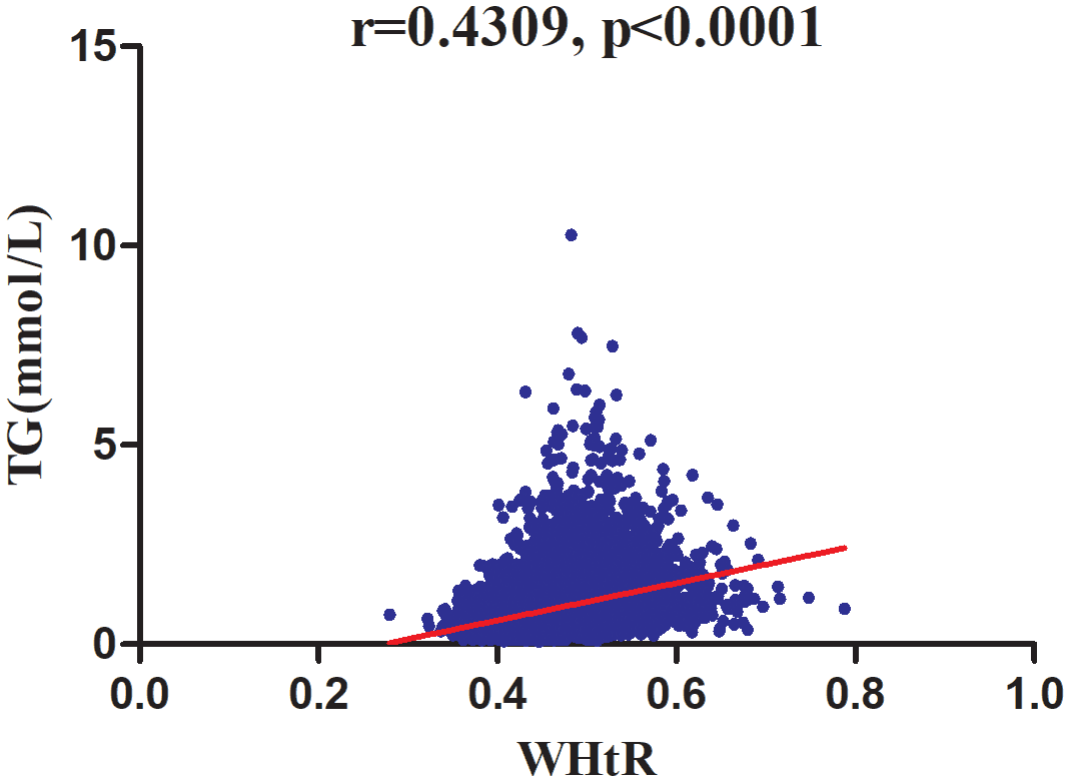
Correlation analysis of WHtR and TG. Correlation analysis results showed that WHtR was positively correlated with TG (r=0.4309, P <0 .0001) (Figure 5).

As shown in **Table 2**, spearman correlation analysis indicated that WHtR was positively correlated with age (r=0.2480, P<0.0001), ethanol consumption (r=0.0856, P<0.0001), BMI (r=0.8385, P<0.0001), WC (r=0.8940, P<0.0001), height (r=0.0285, P=0.0007), ALT (r=0.3685, P<0.0001), AST (r=0.2203, P<0.0001), GGT (r=0.3691, P<0.0001), TC (r=0.2456, P<0.0001), HbA1c (r=0.1928, P<0.0001), FPG (r=0.3130, P<0.0001), SBP (r=0.4104,P<0.0001), DBP (r=0.4053, P<0.0001), males (r=0.2009, P<0.0001), and smoking status (r=0.1209, P<0.0001), but negatively correlated with HDL-c (r=-0.3835, P<0.0001) and regular exercisers (r=-0.0421, P<0.0001). Similarly, TG was positively correlated with age (r=0.2195, P<0.0001), ethanol consumption (r=0.1487, P<0.0001), BMI (r=0.4577, P<0.0001), WC (r=0.4941, P<0.0001), height (r=0.2560, P<0.0001), ALT (r=0.4046, P<0.0001), AST (r=0.1943, P<0.0001), GGT (r=0.4345, P<0.0001), TC (r=0.3847, P<0.0001), HbA1c (r=0.1180, P<0.0001), FPG (r=0.3445, P<0.0001), SBP (r=0.3284,P<0.0001), DBP (r=0.3481, P<0.0001), males (r=0.4044, P<0.0001), and smoking status (r=0.2748, P<0.0001), but negatively correlated with HDL-c (r=-0.5248, P<0.0001) and regular exercisers (r=-0.0208, P=0.0129). There were also significant correlations between WHtR and TG (r=0.4309, P<0.0001) (**Figure 5**).

**Table 2:** Spearman’s correlation of WHtR or TG with potential risk factors of non-alcoholic fatty liver disease.

Variable	Waist-to-height ratio r P	TG r P
**Age, years**	0.2480 <0.0001	0.2195<0.0001
**Ethanol consumption, g/week**	0.0856<0.0001	0.1487<0.0001
**BMI, kg/m^2^**	0.8385<0.0001	0.4577<0.0001
**WC, cm**	0.8940<0.0001	0.4941<0.0001
**WHtR**		0.4309<0.0001
**Height**	0.0285 0.0007	0.2560<0.0001
**ALT, IU/L**	0.3685<0.0001	0.4046<0.0001
**A**S**T, IU/L**	0.2203<0.0001	0.1943<0.0001
**GGT, IU/L**	0.3691<0.0001	0.4345<0.0001
**HDL**-c**, mmol/L**	-0.3835<0.0001	-0.5248 <0.0001
**TC, mmol/L**	0.2456<0.0001	0.3847<0.0001
**TG, mmol/L**	0.4309<0.0001	
**HbA1c, %**	0.1928<0.0001	0.1180<0.0001
**FPG, mmol/L**	0.3130<0.0001	0.3445<0.0001
**SBP, mmHg**	0.4104<0.0001	0.3284<0.0001
**DBP, mmHg**	0.4053<0.0001	0.3481<0.0001
**Male**	0.2009<0.0001	0.4044<0.0001
**Regular exercisers**	-0.0421<0.0001	-0.0208 -0.0208
**Smoking status**	0.1209<0.0001	0.2748<0.0001

ALT, alanine aminotransferase; AST, aspartate aminotransferase; BMI, body mass index; DBP, Diastolic blood pressure; FPG, fasting plasma glucose; GGT, gamma-glutamyl transferase; HbA1c, hemoglobin A1c; HDL-c, high-density lipoprotein cholesterol; SBP, systolic blood pressure; TC, total cholesterol; TG, triglyceride; WC, waist circumference; WHtR, waist-to-height ratio.

### 3.4 The results of univariate analyses using the logistic regression model

The univariate analysis was conducted on the available data, showing that the factor in terms of ethanol consumption was not associated with the risk of NAFLD, but age (OR=1.019, 95%CI 1.014 to 1.024), males (OR=5.018, 95%CI 4.513 to 5.579), BMI (OR=1.646, 95%CI 1.613 to1.680), WC (OR=1.204, 95%CI 1.195 to 1.213), height (OR=1.057, 95%CI 1.052 to1.063), AST (OR=1.090, 95%CI 1.083 to 1.097), ALT (OR=1.103, 95%CI 1.098 to 1.109), GGT (OR=1.040, 95%CI 1.037 to 1.043), TC (OR=1.650, 95%CI 1.570 to 1.734), TG (OR=4.649, 95%CI 4.299 to 5.027), HbA1c (OR=4.415, 95%CI 3.839 to 5.077), FPG (OR=6.907, 95%CI 6.129 to 7.783), SBP (OR=1.053, 95%CI 1.050 to 1.057), DBP (OR=1.079, 95%CI 1.074 to 1.084), ex-smokers (OR=2.214, 95%CI 1.905 to 2.367), and current smokers (OR=1.926, 95%CI 1.733 to 2.139) were positively connected to NAFLD, and HDL-c (OR=0.055, 95%CI 0.047 to 0.064) and regular exercisers (OR=0.816, 95%CI 0.724 to 0.920) were negatively linked with NAFLD (See **Table 3** for detail).

**Table 3:** The results of the univariate analysis.

Variable	Statistics	OR (95%CI) P-value
**Sex**		
Female	6840 (47.997%)	Ref.
Male	7411 (52.003%)	5.018 (4.513, 5.579)<0.0001
**Age, years**	43.533 ± 8.893	1.019 (1.014, 1.024)<0.0001
**Ethanol consumption, g/week**	8.314 ± 46.734	1.000 (0.999, 1.001)0.4693
**BMI, kg/m^2^**	22.065 ± 3.137	1.646 (1.613, 1.680)<0.0001
**WC, cm**	76.185 ± 9.100	1.204 (1.195, 1.213)<0.0001
**Height, cm**	164.796 ± 8.482	1.057 (1.052, 1.063)<0.0001
**WHtR(per 0.1 unit)**	4.623 ± 0.501	16.413 (14.559, 18.502) <0.0001
**ALT, IU/L**	19.764 ± 14.466	1.103 (1.098, 1.109)<0.0001
**GGT, IU/L**	19.126 ± 16.134	1.040 (1.037, 1.043)<0.0001
**AST, IU/L**	18.226 ± 8.669	1.090 (1.083, 1.097)<0.0001
**Regular exercisers**		
No	11781 (82.668%)	Ref.
Yes	2470 (17.332%)	0.816 (0.724, 0.920)0.0008
**HDL-c, mmol/L**	1.459 ± 0.402	0.055 (0.047, 0.064)<0.0001
**TC, mmol/L**	5.124 ± 0.868	1.650 (1.570, 1.734)<0.0001
**TG, mmol/L**	0.891 ± 0.632	4.649 (4.299, 5.027)<0.0001
**HbA1c, %**	.178 ± 0.321	4.415 (3.839, 5.077)<0.0001
**Smoking status**		
Non-smoker	8746 (61.371%)	Ref.
Ex-smoker	2559 (17.957%)	2.124 (1.905, 2.367)<0.0001
Current smoker	2946 (20.672%)	1.926 (1.733, 2.139)<0.0001
**FPG, mmol/L**	5.148 ± 0.412	6.907 (6.129, 7.783)<0.0001
**SBP, mmHg**	113.935 ± 14.822	1.053 (1.050, 1.057)<0.0001
**DBP, mmHg**	71.122 ± 10.383	1.079 (1.074, 1.084)<0.0001

Values are n(%) or mean ± SD or medians (quartiles).

ALT, alanine aminotransferase; AST, aspartate aminotransferase; BMI, body mass index; DBP, diastolic blood pressure; FPG, fasting plasma glucose; GGT, gamma-glutamyl transferase; HbA1c, hemoglobin A1c; HDL-c, high-density lipoprotein cholesterol; SBP, systolic blood pressure; TC, total cholesterol; TG, triglyceride; WC, waist circumference; WHtR, waist-to-height ratio.

OR, odds ratios; CI, confidence, Ref, reference.

### 3.5 Association between WHtR and TG

**Table 4** showed the association of WHtR with TG in the entire cohort. We used a linear regression model to evaluate the associations between WHtR and TG. In the fully adjusted model, WHtR showed a positive association with TG (β= 0.226, 95% confidence interval (CI): 0.206 to 0.247, P<0.0001). That was, for every 0.1 increase in WHtR, TG increased by 0.226mmol/L. We also handled WHtR as a categorical variable for sensitivity analysis and observed the same trend.

**Table 4:** Relationship between WHtR and TG in different models.

Exposure	Non-adjusted (β,95%CI, P)	Adjust I (β,95%CI, P)	Adjust II (β,95%CI, P)
**WHtR(per 0.1 unit)**	0.468 (0.448, 0.487) <0.0001	0.340 (0.320, 0.361) <0.0001	0.226 (0.206, 0.247) <0.0001
**WHtR Tertile**			
**T1**	Ref.	Ref.	Ref.
**T2**	0.251 (0.228, 0.275) <0.0001	0.148 (0.125, 0.171) <0.0001	0.099 (0.077, 0.120) <0.0001
**T3**	0.566 (0.542, 0.589) <0.0001	0.399 (0.375, 0.424) <0.0001	0.271 (0.246, 0.295) <0.0001
**P for trend**	<0.0001	<0.0001	<0.0001

Crude model:we did not adjust other covariants

Model I: we adjusted age, sex, SBP, ethanol consumption, smoking status, and habit of exercise

Model II: we adjusted age, sex, SBP, ALT, AST, GGT, FPG, HbA1c, TC, ethanol consumption, smoking status, and habit of exercise

WHtR,Waist-to-height ratio(per 0.1 unit increase).

β, Regression coefficients; CI, confidence, Ref, reference.

### 3.6 Association between WHtR and NAFLD

The authors constructed three models using the logistic regression model to explore the relationship between the WHtR and NAFLD risk. In the unadjusted model, an increase of 0.1 unit of WHtR was related to a 15.413 times increase in NAFLD risk (OR=16.413, 95%CI 14.559 to 18.502). In the minimally-adjusted model, each additional 0.1 unit of WHtR increased by 15.983 times of NAFLD risk (OR=16.983, 95%CI 14.771 to 19.527). The findings on the link between WHtR and NAFLD obtained from the model were statistically significant. In the fully adjusted model, each additional 0.1 unit of WHtR was accompanied by a 7.743 times increase in the risk of NAFLD (OR=8.743, 95%CI 7.528 to 10.153). The distribution of CI indicates that the model’s link between the WHtR and the risk of NAFLD was reliable (**Table 5**).

**Table 5:** Relationship between TG and NAFLD in different models.

Exposure	Non-adjusted (OR,95%CI, P)	Adjust I (OR,95%CI, P)	Adjust II (OR,95%CI, P)
**TG(mmol/L)**	4.649 (4.299, 5.027) <0.0001	2.195 (2.017, 2.389) <0.0001	1.897 (1.732, 2.078) <0.0001
**TG group**			
**<1.7mmol/L**	Ref.	Ref.	Ref.
**>=1.7mmol/L**	7.488 (6.631, 8.455) <0.0001	2.946 (2.546, 3.409) <0.0001	2.345 (2.000, 2.749) <0.0001

Crude model:we did not adjust other covariants.

Model I: we adjusted age, sex, SBP, BMI, WC, ethanol consumption, smoking status and habit of exercise.

Model II: we adjusted age, sex, BMI, WC, SBP, ALT, AST, GGT, FPG, HbA1c, TC, ethanol consumption, smoking status, and habit of exercise.

OR, odds ratios; CI: confidence, Ref: reference.

A sensitivity analysis was conducted in order to confirm the robustness of the results. Based on the tertiles, we changed the WHtR to a categorical variable and then incorporated the categorically changed WHtR back into the logistic regression equation. According to the results, there was an equidistant trend in the effect sizes (ORs) between groups. The P values for the trend were in agreement with the results obtained with the continuous variable WHtR (**Table 5**).

### 3.7 Association between TG and NAFLD

We also constructed three logistic regression models to explore the relationship between TG and incident NAFLD. In the unadjusted model (Crude model), an increase of 1 mmol/L of TG was connected with a 3.649 times increase in the risk of NAFLD (OR=4.649, 95%CI 4.299 to 5.027). According to the minimally-adjusted model (Model I), when we only considered demographic variables, each additional mmol/L of TG resulted in an increase of 1.195 times of NAFLD risk (OR=2.195, 95%CI 2.017 to 2.389). In the fully adjusted model (Model II), each additional mmol/L of TG was accompanied by an 89.7% increase in NAFLD risk (OR=1.897, 95%CI 1.732 to 2.078). We also transformed the TG into a categorical variable (according to the presence or absence of HTG) and then put it back into the logistic regression equation. After adjusting confounding variables, we found that participants with HTG had 1.345 times increased risk of NAFLD (OR=2.345, 95%CI 2.000 to 2.749). The results suggested that TG was positively associated with NAFLD (**Table 6**).

**Table 6:** Relationship between Waist-to-height ratio and NAFLD in different models.

Exposure	Non-adjusted (OR,95%CI, P)	Adjust I (OR,95%CI, P)	Adjust II (OR,95%CI, P)
**WHtR(per 0.1 unit)**	16.413 (14.559, 18.502) <0.0001	16.983 (14.771, 19.527) <0.0001	8.743 (7.528, 10.153) <0.0001
**WHtR Tertile**			
**T1**	Ref.	Ref.	Ref.
**T2**	11.188 (8.494, 14.736) <0.0001	8.529 (6.453, 11.272) <0.0001	5.655 (4.244, 7.534) <0.0001
**T3**	53.971 (41.301, 70.529) <0.0001	38.975 (29.635, 51.259) <0.0001	17.354 (13.079, 23.025) <0.0001
**P for trend**	<0.0001	<0.0001	<0.0001

Crude model:we did not adjust other covariants.

Model I: we adjusted age, sex, SBP, ethanol consumption, smoking status and habit of exercise.

Model II: we adjusted age, sex, SBP, ALT, AST, GGT, FPG, HbA1c, TC, TG, ethanol consumption, smoking status, and habit of exercise.

WHtR,Waist-to-height ratio(per 0.1 unit increase).

OR, odds ratios; CI: confidence, Ref: reference.

### 3.8 Mediated effect of TG on the association between WHtR and NAFLD

It was found that WHtR and TG were positively associated with NAFLD, while TG was positively associated with WHtR, pointing to TG as a mechanistic link between WHtR and NAFLD. We performed a mediation analysis to determine the role of TG in mediating the association between WHtR and NAFLD in order to examine their internal relationships.

**Figure 6:**
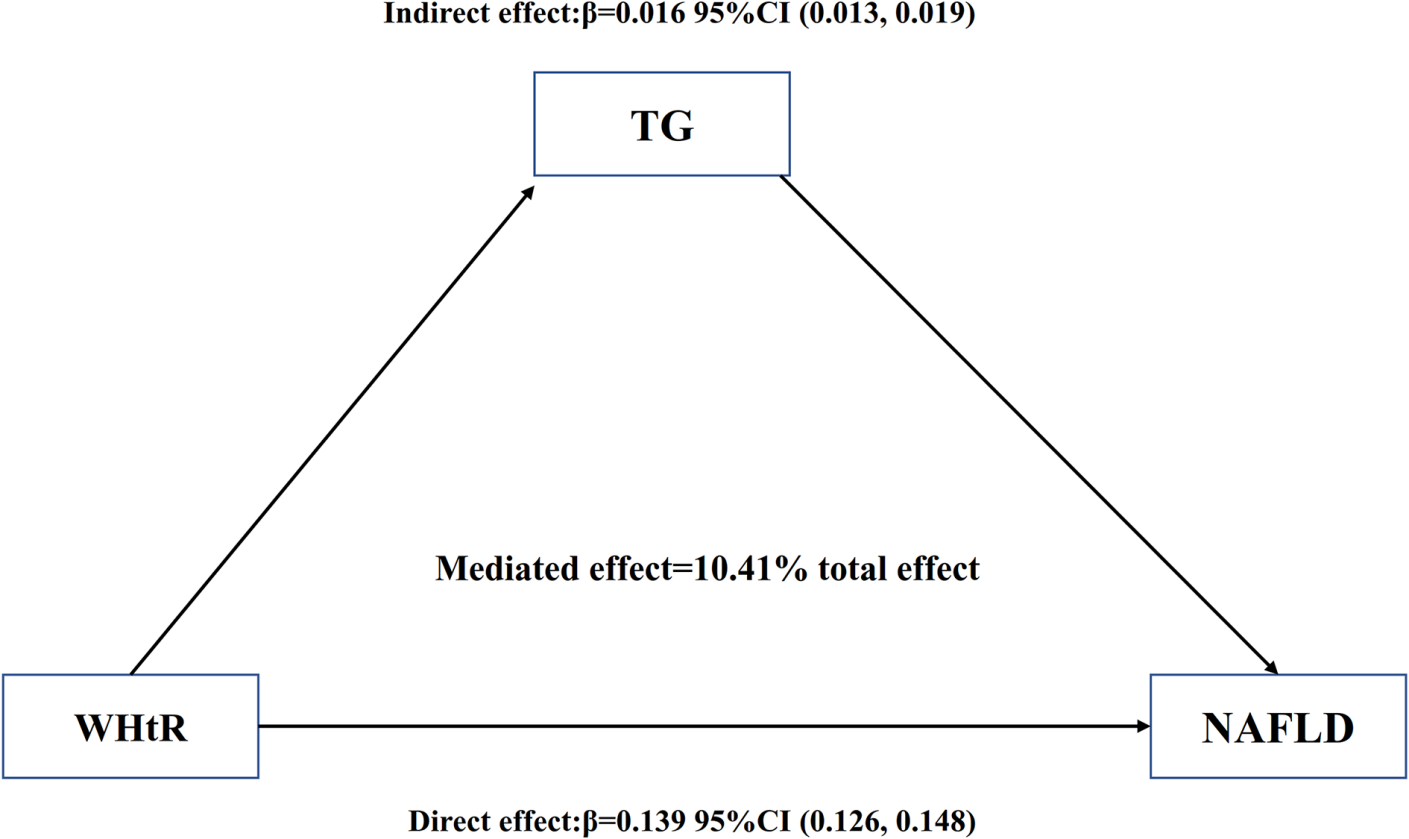
Mediation of TG on the association between WHtR and NAFLD. Figure 6 indicated that WHtR had a significant direct effect on NAFLD(β=0.139, 95%CI: 0.126, 0.148), and TG partly mediated the indirect effect of WHtR on NAFLD(β=0.016, 95% CI: 0.013-0.019). Therefore, approximately 10.41% of the WHtR effect on NAFLD was mediated through TG levels. Zero was not included in 95% confidence intervals representing statistical significance.

As shown in **Table 7** and **Figure 6**, mediation analysis indicated that WHtR had a significant direct effect on NAFLD (β=0.139, 95%CI: 0.126, 0.148), and TG partly mediated the indirect effect of WHtR on NAFLD (β=0.016, 95% CI: 0.013-0.019). Therefore, approximately 10.41% of the WHtR effect on NAFLD was mediated through TG levels. The above results were obtained after adjusting for factors such as age, sex, SBP, ALT, AST, GGT, FPG, HbA1c, TC, ethanol consumption, smoking status, and habit of exercise. The results were consistent with the above results when no confounding factors or only some demographic variables were adjusted.

**Table 7:** Mediation analysis of the association between WHtR and the risk of NAFLD mediated by TG.

Exposure	Non-adjusted β (95%CI)P-value	Adjust I β (95%CI) P-value	Adjust II β(95%CI) P-value
**Direct effect**	0.187 (0.178, 0.196) <0.0001	0.177 (0.168, 0.186) <0.0001	0.139 (0.126, 0.148) <0.0001
**Indirect effect**	0.050 (0.045, 0.055) <0.0001	0.031 (0.027, 0.035) <0.0001	0.016 (0.013, 0.019) <0.0001
**Total effect**	0.237 (0.229, 0.244) <0.0001	0.208 (0.199, 0.216) <0.0001	0.155 (0.142, 0.165) <0.0001
**PM, %**	21.10	14.83	10.41
**P-value**	<0.0001	<0.0001	<0.0001

Crude model: we did not adjust other covariants.

Model I: we adjusted age, sex, SBP, ethanol consumption, smoking status and habit of exercise.

Model II: we adjusted age, sex, SBP, ALT, AST, GGT, FPG, HbA1c, TC, ethanol consumption, smoking status, and habit of exercise.

PM, percent mediation.

### 3.9 The results of subgroup analyses

**Table 8** showed that the mediating effects of TG on the association between WHtR and NAFLD were present in most of the subgroup populations. However, in the participants with age<30 years,

**Table 8:** Mediation analysis of the association between WHtR and NAFLD mediated by TG in prespecified and exploratory subgroups.

Characteristic	No. of participants	Total effect	NDE	NIE	PM, %	P-value
**Age, years** <30 30 to 40 40 to 50 50 to 60 ≥60	401 4885 5278 3052 635	0.066 (0.026, 0.099) 0.141 (0.121, 0.154) 0.133 (0.119, 0.147) 0.160 (0.141, 0.180) 0.132 (0.092, 0.170)	0.062 (0.024, 0.093) 0.124 (0.105, 0.138) 0.119 (0.105, 0.133) 0.145 (0.127, 0.164) 0.124 (0.086, 0.161)	0.004 (-0.0002, 0.012) 0.017 (0.012, 0.021) 0.014 (0.010, 0.018) 0.015 (0.010, 0.020) 0.008 (0.0006, 0.017)	5.61 11.83 10.62 9.24 5.84	0.0680 <0.0001 <0.0001 <0.0001 0.0240
**Sex**						
Male	7411	0.191 (0.177, 0.204)	0.173 (0.159, 0.186)	0.018 (0.015, 0.022)	9.48	<0.0001
Female	6840	0.109 (0.095, 0.119)	0.097 (0.084, 0.107)	0.012 (0.009, 0.015)	11.07	<0.0001
**Regular exercisers**						
Yes	2470	0.151 (0.130, 0.170)	0.136 (0.115, 0.157)	0.015 (0.009, 0.022)	9.61	<0.0001
No	11781	0.155 (0.141, 0.165)	0.139 (0.125, 0.148)	0.016 (0.014, 0.019)	10.64	<0.0001
**SBP, mmHg**						
<140	13596	0.149 (0.137, 0.159)	0.133 (0.121, 0.143)	0.016 (0.013, 0.019)	10.77	<0.0001
≥140	655	0.198 (0.157, 0.239)	0.185 (0.147, 0.225)	0.013 (0.004, 0.024)	6.75	0.0020
**DBP, mmHg**						
<90	13617	0.149 (0.136, 0.159)	0.133 (0.120, 0.143)	0.016 (0.013, 0.019)	10.71	<0.0001
≥90	634	0.202 (0.164, 0.243)	0.190 (0.153, 0.229)	0.012 (0.004, 0.023)	5.96	0.0060
**Smoking status**						
Non-smoker	8746	0.139 (0.124, 0.150)	0.123 (0.108, 0.134)	0.016 (0.013, 0.019)	11.43	<0.0001
Ex-smoker	2559	0.177 (0.156, 0.201)	0.158 (0.137, 0.182)	0.019 (0.013, 0.026)	10.56	<0.0001
Current smoker	2946	0.173 (0.151, 0.194)	0.159 (0.137, 0.180)	0.014 (0.009, 0.020)	8.26	<0.0001
**BMI**
<25kg/m^2^	11987	0.085 (0.075, 0.092)	0.073 (0.064, 0.080)	0.012 (0.009, 0.014)	13.48	<0.0001
≥25kg/m^2^	2264	0.099 (0.077, 0.122)	0.097 (0.075, 0.120)	0.002 (-0.002, 0.006)	2.06	0.3260
**Ethanol consumption, g/week**						
=0	4735	0.157 (0.138, 0.171)	0.143 (0.125, 0.157)	0.014 (0.011, 0.018)	9.10	<0.0001
>0	9516	0.149 (0.138, 0.160)	0.132 (0.121, 0.143)	0.017 (0.014, 0.020)	11.26	<0.0001

Note 1:Above model adjusted for age, sex, SBP, ALT, AST, GGT, FPG, HbA1c, TC, ethanol consumption, smoking status, and habit of exercise.

Note 2:In each case, the model is not adjusted for the stratification variable when the stratification variable was a categorical variable.

DBP: Diastolic blood pressure; SBP: Systolic blood pressure; BMI, body mass index.

PM, percent mediation; NDE, natural direct effect; NIE, natural indirect effect.

although TG accounted for 5.61% of WHtR-related NAFLD, the mediation effects seemed to be insignificant (P=0.0680). Similarly, the mediating effect of TG on the association between WHtR and NAFLD was also insignificant in the populations with BMI≥25kg/m^2^ (P=0.3260).

## 4 Discussion

The cross-sectional study was designed to investigate the associations of NAFLD with WHtR and TG and to determine whether TG mediates the effect of WHtR on NAFLD. We found that both WHtR and TG were positively associated with NAFLD, and WHtR was positively associated with TG. Mediation analysis indicated that WHtR had a significant direct effect on NAFLD (β=0.139, 95%CI: 0.126, 0.148), and TG partly mediated the indirect effect of WHtR on NAFLD (β=0.016, 95% CI: 0.013-0.019). Approximately 10.41% of the WHtR effect on NAFLD was mediated through TG levels.

NAFLD prevalence was 17.59% in this study, which was relatively low compared to Asian prevalence (29.62%) (7). The cause may be related to participants with type 2 diabetes, and FPG≥ 6.1mmol/L were excluded from this study. Impaired fasting glucose (IFG) was defined as FPG of at least 6.1 mmol/L and less than 7.0 mmol/L (40). Studies have shown that IFG and type 2 diabetes are closely related to the development of hepatic IR and NAFLD (41, 42). A significant portion of participants still maintains their habit of exercising (17.33%). Regular exercise can reduce the risk of NAFLD and is currently the primary means of preventing NAFLD occurrence and controlling NAFLD development (43). Based on the above, it is not doubtful that the prevalence of NAFLD in our study population is lower compared with that of Asian people.

According to numerous studies, NAFLD incidence increases with obesity, which supports the notion that NAFLD is an obesity-related condition (7). Our findings are consistent with previous research, which found that TG increases with increasing WHtR (44) and, intriguingly, is also associated with NAFLD (17–19). The present study found that both WHtR and TG were positively associated with NAFLD, while WHtR was positively associated with TG, indicating a mechanistic link between WHtR and NAFLD, which may be explained by TG. Clinical trials, however, have failed to demonstrate that TG is a mediator of the relationship between WHtR and NAFLD development. However, we found that TG accounted for 10.41% of the relationship between WHtR and NAFLD, based on **Tables 7** and **Table 8**, suggesting that part of the effect of WHtR on NAFLD is mediated through TG. The mediation analysis results may be explained by insulin resistance and triglyceride accumulation. It has been suggested that IR may play a role in obesity and hypertriglyceridemia in some previous studies (45, 46). Additionally, IR is widely recognized as the main cause of NAFLD (47), and WHtR and HTG are directly related to IR (48, 49), as a result of which NAFLD develops and progresses. The factors mentioned above may indicate incomplete associations between WHtR, TG, and NAFLD. Furthermore, as insulin resistance increases, the lipid metabolism changes due to peripheral lipolysis, an increase in TG synthesis, and an increase in hepatic fatty acid uptake. All of these factors may increase the amount of hepatocellular TG (50), which histologically and metabolically is generally accepted as one of the hallmark features of NAFLD (51). It has been suggested that obesity contributes to the accumulation of TG (20). We, therefore, propose that the mediation of the relationship between WHtR and NAFLD by TG may ultimately be accomplished through insulin resistance and hepatic triglyceride accumulation.

By examining the relationship among WHtR, TG, and NAFLD, we found that 10.41% of the WHtR effect on NAFLD is accomplished through TG mediation. These findings have important clinical implications. On the one hand, it provides reference evidence to clarify the specific mechanism and pathway of WHtR on NAFLD. On the other hand, it provides a new way to prevent the occurrence and progression of NAFLD clinically. Clinically we can prevent and improve NAFLD by controlling body weight. Meanwhile, we can also actively control serum triglyceride levels to attenuate NAFLD risk due to weight gain. It needs to be further pointed out that other unknown factors may be more responsible for mediating the association between WHtR and NAFLD for the additional 89.59%.

Subgroup analysis results suggested that TG’s mediating effect on the association between WHtR and NAFLD was insignificant in the populations with BMI≥25kg/m^2^ (P=0.3260). The results of our further analysis found that the association strength of WHtR with NAFLD was strengthened when BMI<25 kg/m^2^ (OR=10.895, 95%CI 8.675-13.683), but weakened when BMI≥25kg/m^2^ (OR=2.846, 95%CI 2.148-3.770), (P for interaction<0.0001) (**Table S1**). The reason was that other variables at participants’ baselines may also have affected the risk of NAFLD. It could be found that compared with the BMI<25kg/m^2^ group, people with BMI≥25kg/m^2^ have generally higher levels or proportion of age, males, ALT, GGT, AST, TC, FPG, SBP, DBP, HbA1c, ex-smokers and current smokers (**Table S2**). However, most of the above indicators were closely related to NAFLD (52–57). Due to the presence of these NAFLD risk factors when BMI is higher than 25kg/m^2^, the WHtR has a relatively weak impact on NAFLD risk. Then, when BMI was below 25kg/m^2^, the level of NAFLD risk factors, such as GGT, SBP, ALT, TC, FPG, and HbA1c, was lower, and the impact on NAFLD was weakened; at this time, the effect of WHtR was relatively enhanced. Since the association between WHtR and NAFLD was weakened when BMI was above 25 kg/m^2^, the mediating effect of TG on the relationship between WHtR and NAFLD was correspondingly attenuated to 2.06% at this time, making this result not statistically significant. The findings provide an essential reference for preventing NAFLD by intervening with WHtR and TG in the clinical. Clinically, although the BMI of patients is less than 25kg/m^2^, we still need to control the body weight and serum triglyceride levels actively from both the direct and indirect effect viewpoints to reduce the risk of NAFLD.

There are some strengths of our study, which are listed below (1). One strength of our study is the large sample size that allows such analysis (2). This is the first time to explore the contribution of TG as a mediator factor to the relationship between WHtR and NAFLD (3). By using different statistical methods, we examined the internal relationship between WHtR, TG, and NAFLD. This strengthened our understanding of their relationship (4). We conducted sensitivity analyses (target independent variable transformations, subgroup analyses) to assess the robustness of the findings.

There are a few shortcomings in our research that should be noted. First, in the current study, NAFLD was diagnosed by ultrasonography with no histological confirmation, rather than liver biopsy, the gold standard for diagnosing NAFLD. Despite its limitations, however, ultrasonography is a popular method for detecting NAFLD due to its safety, availability, and economics. In addition, the diagnosis of NAFLD was based on different gastroenterologists. However, different experts got different thinking of NAFLD. Therefore, the diagnostic consistency of different experts could not be fully guaranteed. The original study did not provide information on the consistency of the diagnosis of NAFLD among different experts. In the future, we can design our studies to conduct a consistent evaluation of the results of NAFLD diagnosed by different experts to ensure consistency of results. Second, this retrospective observational study provided association inference rather than establishing a causal relationship between the WHtR and NAFLD risk. However, this study avoided observational bias since it was a retrospective study. Moreover, the results drawn from this study were based on a large sample and can therefore be considered reliable. Third, due to this study being a secondary analysis of previous research (33), type 2 diabetes patients and persons with FPG≥6.1mmol/L were not included in the data package, so it might cause a particular selection bias. From another viewpoint, type 2 diabetes and NAFLD have been linked positively in numerous studies in the past (41, 42, 58), but this study still found a positive association between WHtR and NAFLD, even after excluding patients with type 2 diabetes and IFG. This association can therefore be considered relatively reliable. Fourth, TG, WHtR, and NAFLD were strongly associated with other factors (LDL, BMI, smoking, SBP, etc.). Therefore, the relationship between WHtR and NAFLD cannot be solely mediated by the TG. Other factors (LDL, BMI, smoking, SBP, etc.) should also mediate the relationship between waist-to-height ratio and NAFLD. In the future, we can analyze in depth the role of LDL, BMI, smoking, SBP, etc., in mediating the relationship between WHtR and NAFLD simultaneously.

## 5 Conclusion

WHtR is positively associated with NAFLD, and TG partly mediated the association between WHtR and NAFLD in a Japanese population. This finding indicates that we should pay more attention to WHtR and TG levels due to their effect on NAFLD risk. It provides a new way to prevent the occurrence and progression of NAFLD clinically. Clinically, we can prevent and improve NAFLD by controlling body weight. Meanwhile, we can also actively control serum triglyceride levels to attenuate NAFLD risk due to weight gain.

## Data availability statement

The original contributions presented in the study are included in the article/supplementary materials. Further inquiries can be directed to the corresponding authors.

## Ethics statement

The studies involving human participants were reviewed and approved by the ethics committee of Murakami Memorial Hospital. The patients/participants provided their written informed consent to participate in this study.

## Author contributions

HH, YH, and YL contributed to the study design, and drafted the manuscript. HH and MG are responsible for statistical analysis, research, and interpretation of the data. They are responsible for the integrity of the data and the accuracy of the data analysis. MG contributed to the discussion and reviewed the manuscript. QW revised the manuscript and designed the study. QW and HH are the guarantors of this work. All authors read and approved the final manuscript.

## Funding

This study was supported by the Shenzhen Key Medical Discipline Construction Fund (SZXK009), and the Discipline Construction Ability Enhancement Project of the Shenzhen Municipal Health Commission (SZXJ2017031).

## Acknowledgments

As this is a secondary analysis, the data and method descriptions are primarily derived from the following studies: Okamura T, Hashimoto Y, Hamaguchi M, Obora A, Kojima T, Fukui M. Ectopic fat obesity presents the greatest risk for incident type 2 diabetes: a population-based longitudinal study. Int J Obes (Lond). 2019 Jan;43 (1):139-148. doi: 10.1038/s41366-018-0076-3. Epub 2018 May 1. PMID: 29717276^24^. We are grateful to all the authors of the study.

## Conflict of interest

The authors declare that the research was conducted in the absence of any commercial or financial relationships that could be construed as a potential conflict of interest.

## Publisher’s note

All claims expressed in this article are solely those of the authors and do not necessarily represent those of their affiliated organizations, or those of the publisher, the editors and the reviewers. Any product that may be evaluated in this article, or claim that may be made by its manufacturer, is not guaranteed or endorsed by the publisher.

## Abbreviations

WHtR, waist-to-height ratio; FPG, fasting plasma glucose; ALT, alanine aminotransferase; TG, triglyceride; NAFLD, non-alcoholic fatty liver disease; AST, aspartate aminotransferase; BMI, body mass index; NASH, non-alcoholic steatohepatitis; HTG, hypertriglyceridemia; DBP, diastolic blood pressure; NAGALA, NAfld in the Gifu Area, Longitudinal Analysis; IR, insulin resistance; GGT, gamma-glutamyl transferase; HbA1c, hemoglobin A1c; SBP, systolic blood pressure; HDL-c, high-density lipoprotein cholesterol; WC, waist circumference; OR, odds ratio; Ref, reference; CI, confidence intervals; TC, total cholesterol; IFG, impaired fasting glucose; SD, standard deviation.
