# Supplementary material for: Triglyceride: A mediator of the association between waist-to-height ratio and non-alcoholic fatty liver disease: A second analysis of a population-based study
Source: Front Endocrinol (Lausanne). 2022 Oct 31;13:973823. doi: 10.3389/fendo.2022.973823 (PMC9659645; doi:10.3389/fendo.2022.973823)
Supplement: Supplementary file 1 [file Table_1.docx]

**Triglyceride: a mediator of the association between waist-to-height ratio and non-alcoholic fatty liver disease: a second analysis of population-based cohort study**

**Running title:** **WHtR, TG and NAFLD**

**Haofei Hu^1,2,7#^, Yong Han^3,4,7#^,** **Yufei Liu^5,6,7#^,Mijie Guan^1,2,7*^,Qijun Wan^1,2,7*^**

^1^Department of Nephrology, Shenzhen Second People’s Hospital, Shenzhen 518000, Guangdong Province, China

^2^Department of Nephrology, The First Affiliated Hospital of Shenzhen University, Shenzhen 518000, Guangdong Province, China

^3^Department of Emergency, Shenzhen Second People’s Hospital, Shenzhen 518000, Guangdong Province, China

^4^Department of Emergency, The First Affiliated Hospital of Shenzhen University, Shenzhen 518000, Guangdong Province, China

^5^Department of Neurosurgery, Shenzhen Second People’s Hospital, Shenzhen 518000, Guangdong Province, China

^6^Department of Neurosurgery, The First Affiliated Hospital of Shenzhen University, Shenzhen 518000, Guangdong Province, China

^7^Shenzhen University Health Science Center, Shenzhen 518000, Guangdong Province, China

**^#^Haofei Hu, Yong Han and Yufei Liu have contributed equally to this work.**

***Corresponding author**

**Mijie Guan**

Department of Nephrology,

Shenzhen Second People’s Hospital,

No.3002 Sungang Road, Futian District,

Shenzhen 518000,

Guangdong Province,

China

Tel:+86-755-83366388

E-mail: [857134191@qq.com](mailto:huhaofei0319@126.com)

**Qijun Wan**

Department of Nephrology,

Shenzhen Second People’s Hospital,

No.3002 Sungang Road, Futian District,

Shenzhen 518000,

Guangdong Province,

China

Tel:+86-755-83366388

E-mail: [wanqijun12345@126.com](mailto:huhaofei0319@126.com)

**Table S1. Effect Modification of BMI on the association between WHtR and NAFLD risk**

| NAFLD | Crude model  OR (95%CI) P-value | Model I  OR (95%CI) P-value | Model II  OR (95%CI) P-value |
| --- | --- | --- | --- |
| BMI group |  |  |  |
|  |  |  |  |
| <25 kg/m^2^ | 15.080 (12.622, 18.017) <0.0001 | 0.984 (0.981, 0.987) <0.0001 | 10.895 (8.675, 13.683)<0.0001 |
| >=25 kg/m^2^ | 2.925 (2.323, 3.683) <0.0001 | 0.989 (0.986, 0.992) <0.0001 | 2.846 (2.148, 3.770) <0.0001 |
| P value for interaction | <0.0001 | <0.0001 | <0.0001 |

Crude model:we did not adjust other covariants

Model I: we adjusted age, sex, SBP, ethanol consumption, smoking status and habit of exercise

Model II: we adjusted age, sex, SBP, ALT, AST, GGT, FPG, HbA1c, TC, TG, ethanol consumption, smoking status, and habit of exercise

WHtR,Waist-to-height ratio(per 0.1 unit increase)

OR, odds ratios; CI: confidence, Ref: reference;

## TableS2. The Baseline Characteristics of participants in different BMI groups.

| BMI(kg/m^2^) | <25 | >=25 | P-value |
| --- | --- | --- | --- |
| Participants | 11987 | 2264 |  |
| Age, years | 43.45 ± 8.99 | 43.95 ± 8.38 | 0.015 |
| Ethanol consumption, g/week | 1.00 (0.00-36.00 | 1.00 (0.00-54.00) | <0.001 |
| WC, cm | 73.78 ± 7.29 | 88.93 ± 6.84 | <0.001 |
| Height, cm | 164.35 ± 8.41 | 167.16 ± 8.47 | <0.001 |
| WHtR | 0.45 ± 0.04 | 0.53 ± 0.04 | <0.001 |
| ALT, IU/L | 16.00 (12.00-21.00) | 25.00 (18.00-36.00) | <0.001 |
| GGT, IU/L | 14.00 (11.00-19.00) | 21.00 (15.00-32.00) | <0.001 |
| AST, IU/L | 17.00 (14.00-20.00) | 20.00 (16.00-25.00) | <0.001 |
| HDL-c, mmol/L | 1.51 ± 0.40 | 1.20 ± 0.29 | <0.001 |
| TC, mmol/L | 5.07 ± 0.86 | 5.39 ± 0.88 | <0.001 |
| TG, mmol/L | 0.67 (0.46-0.98) | 1.15 (0.79-1.66) | <0.001 |
| HbA1c, % | 5.16 ± 0.31 | 5.27 ± 0.34 | <0.001 |
| FPG, mmol/L | 5.11 ± 0.41 | 5.37 ± 0.37 | <0.001 |
| SBP, mmHg | 111.82 ± 13.89 | 125.12 ± 14.57 | <0.001 |
| DBP, mmHg | 69.68 ± 9.80 | 78.75 ± 10.07 | <0.001 |
| Male, n(%) | 5760 (48.05%) | 1651 (72.92%) | <0.001 |
| Regular exercisers, n(%) | 2135 (17.81%) | 335 (14.80%) | <0.001 |
| Smoking status, n(%) |  |  | <0.001 |
| Non-smoker | 7613 (63.51%) | 1133 (50.04%) |  |
| Ex-smoker | 2056 (17.15%) | 503 (22.22%) |  |
| Current smoker | 2318 (19.34%) | 628 (27.74%) |  |

Values are n(%) or mean±SD or medians (quartiles)

ALT, alanine aminotransferase; AST, aspartate aminotransferase; BMI, body mass index; DBP, Diastolic blood pressure; FPG, fasting plasma glucose; GGT, gamma-glutamyl transferase; HbA1c, hemoglobin A1c; HDL-c, high-density lipoprotein cholesterol; SBP, Systolic blood pressure; TC, Total cholesterol; TG, Triglyceride; WC, waist circumference;

**Figure S1. Comparison of WHtR and TG between NAFLD and non-NAFLD participants.**

Figure S1 indicated that both TG and WHtR levels were higher in individuals with NAFLD than in those without NAFLD.
