# Supplementary figures and images for: Triglyceride: A mediator of the association between waist-to-height ratio and non-alcoholic fatty liver disease: A second analysis of a population-based study
Source: Front Endocrinol (Lausanne). 2022 Oct 31;13:973823. doi: 10.3389/fendo.2022.973823 (PMC9659645; doi:10.3389/fendo.2022.973823)

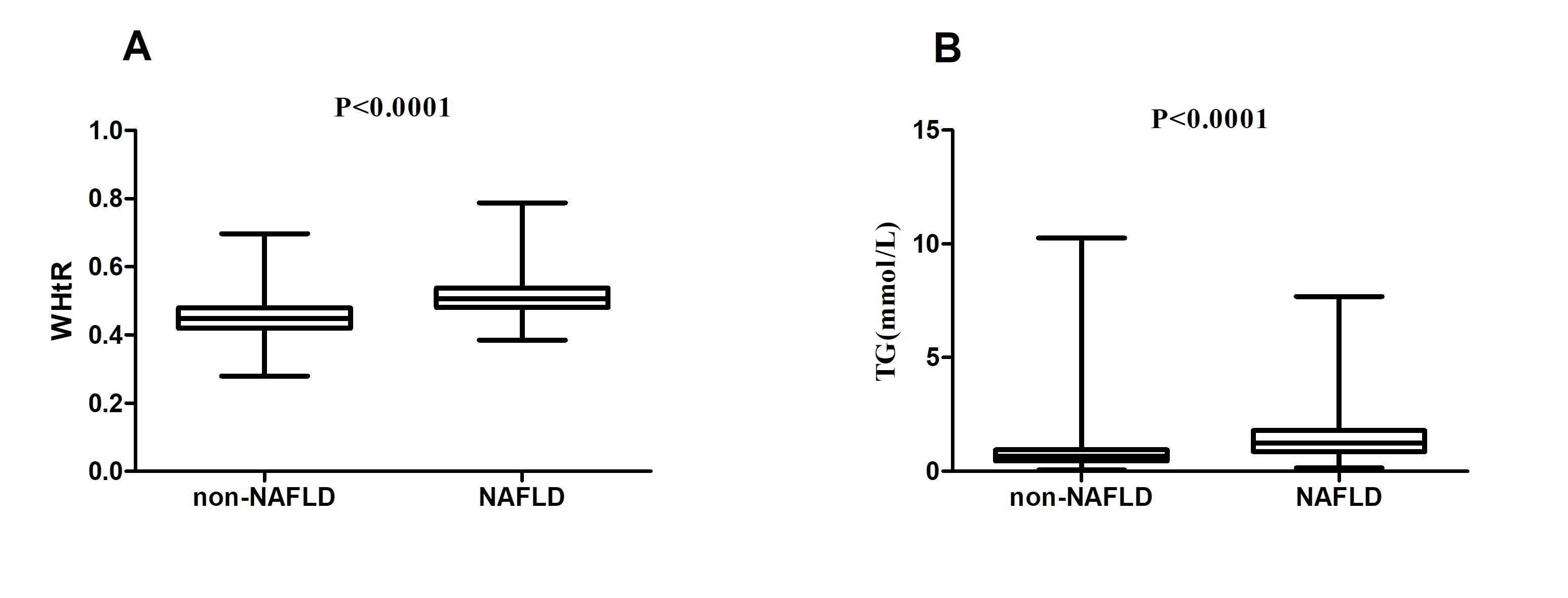

Supplement: Supplementary file 3 [file Image_1.tif]
